# Interaction Effects between Organochlorine Pesticides and Isoflavones *In Vitro* and *In Vivo*

**DOI:** 10.1155/2016/6861702

**Published:** 2016-08-15

**Authors:** Yunbo Zhang, Jipeng Guo, Xiao Zhang, Jingjing Guo, Ming Zhang, Yang Yang, Xiaolin Na

**Affiliations:** Department of Environmental Hygiene, Public Health College, Harbin Medical University, Harbin, Heilongjiang 150081, China

## Abstract

Organochlorine pesticides (OCPs) have caused increasing global concern due to their high toxicity, persistence, bioaccumulation, and significant adverse effects on human health. This study was to explore the interaction effects between OCPs and isoflavones. Six kinds of OCPs and 2 kinds of isoflavones-genistein and daidzein were included to study their effect on MCF-7 cells* in vitro*. Eighty-one female Sprague-Dawley rats were randomized to 9 groups according to factorial design to study the interaction effect between isoflavones and *γ*-HCH. Compared to organochlorine pesticides alone group, proliferation rate of MCF-7 cells was lower in 100 *μ*mol/L genistein + organochlorine pesticides and 100 *μ*mol/L daidzein + organochlorine pesticides group (*p* < 0.05).* In vivo* study showed that there are interaction effects on kidney weight and liver weight when treated with isoflavones and *γ*-HCH. The changes in uterine morphology and positive expression of ER*α* showed inhibition effects between isoflavones and *γ*-HCH. In conclusion, the data suggests that there are interactions between isoflavones and OCPs* in vitro* and* in vivo*.

## 1. Introduction

The research on endocrine-disrupting chemicals (EDCs) has attracted more and more interest of scientists. Organochlorine pesticides (OCPs), which are one of the EDCs, can be lipophilic, stable, difficult to be degraded, volatile, and accumulative. They are easy to be accumulated in environment, and humans can absorb them via food chain enrichment through the digestive tract, respiratory tract, and skin [1, 2]. The OCPs are usually divided into three main groups including DDT and its derivatives (DDTs and DDEs), HCHs, and chlorinated cyclodiene such as chlordane. DDEs are the main bioaccumulative metabolite of the organochlorine insecticide DDT. Exposure of men or animals to OCPs can cause some side effects including reproductive toxicity and even cancer [3, 4].

Soybean isoflavones are a kind of plant estrogen, extracted in soybean, mainly including genistein and daidzein. Genistein is the most frequently studied isoflavone due to its biological activities of improving cardiovascular, bone, and postmenopausal health. Daidzein, a major component of soy with structural similarities to estrogen, can exert effects of anti-inflammatory, lowering lipid levels and increasing mitochondrial biogenesis. Many studies have indicated that the isoflavones or single components (genistein or daidzein) have many important biological activities; for example, they can improve the women's menopause syndrome and prevent osteoporosis. Messina et al. found that higher isoflavones intake was associated with 25% reduction in recurrence in 9,514 breast cancer survivors over 7.4-year follow-up period [5]. Moreover, although the antiproliferation effect in different breast tumor cell types treated with different concentration of genistein and daidzein varied, their effects were related to ER*α* and c-erbB-2 expression [6].

Breast cancer is the most common and leading type of cancer in women, and the survival rates are very poor in developing countries. Human breast cancer cell line MCF-7 was used as the hormone-responsive breast cancer cell line in many studies. Estrogen can make the pathological progress of mammary gland cells accelerate, which is a major cause of estrogen dependent breast cancer. A large number of studies showed that OCPs could combine with estrogen receptor to cause some side effects including reproductive toxicity [7, 8]. According to Shekhar, p,p′-DDT could evoke responses and further enhance the responses together with estradiol or o,p′-DDT in estrogen receptor-positive breast cancer cells [9]. However, studies showed that both genistein and DDT can be combined with estrogen receptor. High concentrations of genistein can inhibit the proliferation of cells while DDTs promote the proliferation of cells, although the effects of isoflavones on breast cancer remain controversial and human clinical investigations are needed [10]. We can still predict that more phytoestrogen dietary intake may drop the weakness of exogenous estrogen.

OCPs affect human health mainly by continuous exposures at low dose [11]. And human can be exposed to isoflavones by daily food. It has practical significance to carry out research on interactions between OCPs and isoflavones.

## 2. Material and Methods

### 2.1. *In Vitro* Experiment

#### 2.1.1. Chemicals

*β*-HCH, *γ*-HCH, o,p′-DDT, p,p′-DDT, p,p′-DDE, and chlordane were purchased from Dr. Ehrenstorfer GmbH Ltd. Co. (Germany). Genistin (Gen, purity ≥ 99%), daidzein (Dai, purity ≥ 99%), estradiol (E_2_), and methyl thiazolyl tetrazolium (MTT) were purchased from Sigma Chemical Co. (St. Louis, MO).

#### 2.1.2. Cell Culture and Treatment

MCF-7 cells were cultured in a 37°C, 5% CO_2_ saturated humidity incubator with DMEM (containing 100 IU/mL penicillin/streptomycin and 10% FBS). The cells had digestive transfer culture after being grown to 80% cell density. Discard DMEM and rinse it two times by PBS (pH 7.4) solution. 0.05% trypsin (1 mL) was added to cover cell layer for 2-3 minutes. After discarding dispersed liquid, 5 mL DMEM was added in culture bottle. Cells were made into single cell suspension and placed into culture flask at 37°C.

#### 2.1.3. MTT Experiment

MCF-7 cells were cultured in phenol red-free DMEM (Sigma-Aldrich) with 100 IU/mL penicillin/streptomycin and 10% charcoal stripped estrogen-free FBS (Invitrogen). Cells were treated with the following solutions: (i) DMEM medium alone (the control group); (ii) 100 *μ*mol/L genistein group; (iii) 100 *μ*mol/L daidzein group; (iv) 1 *μ*mol/L o,p′-DDT, 1 *μ*mol/L p,p′-DDT, 1 *μ*mol/L *γ*-HCH, 1 *μ*mol/L chlordane, 10 *μ*mol/L p,p′-DDE, and 20 *μ*mol/L *β*-HCH; (v) 100 *μ*mol/L genistein + 1 *μ*mol/L o,p′-DDT, 100 *μ*mol/L genistein + 1 *μ*mol/L p,p′-DDT, 100 *μ*mol/L genistein + 1 *μ*mol/L *γ*-HCH, 100 *μ*mol/L genistein + 1 *μ*mol/L chlordane, 100 *μ*mol/L genistein + 10 *μ*mol/L p,p′-DDE, and 100 *μ*mol/L genistein + 20 *μ*mol/L *β*-HCH; (vi) 100 *μ*mol/L daidzein + 1 *μ*mol/L o,p′-DDT, 100 *μ*mol/L daidzein + 1 *μ*mol/L p,p′-DDT, 100 *μ*mol/L daidzein + 1 *μ*mol/L *γ*-HCH, 100 *μ*mol/L daidzein + 1 *μ*mol/L chlordane, 100 *μ*mol/L daidzein + 10 *μ*mol/L p,p′-DDE, and 100 *μ*mol/L daidzein + 20 *μ*mol/L *β*-HCH. Cell growth and inhibition rate was measured by 3-(4,5-dimethylthiazol-2-yl)-2,5-diphenyltetrazolium bromide (MTT) assay.

Cells were seeded in 96-well plates (cell density of 3 × 10^4^/mL) for 24 h and exposed to various concentrations of genistein, daidzein, or OCPs for 48 h. Then the cells were incubated with 20 *μ*L 5 mg/mL MTT in the dark for 4 h at 37°C. Absorbance values (optical density, OD) were obtained using a microplate reader (model 680: Bio-Rad Laboratories, Inc., Hercules, CA, USA) at a wavelength of 490 nm. Wells containing cells in DMEM were served as the normal control. To convert OD values to proliferation rate, the following equation was used: proliferation rate (PR) = OD of test concentration/OD of control *∗* 100%. Each concentration was measured in 3–5 repeated wells, and every assay was tested ≥3 times.

### 2.2. *In Vivo* Experiment

#### 2.2.1. Chemical

Isoflavones were purchased from Rongsheng Ltd. Co. (Xi'an, China) with purity ≥98%. *γ*-HCH was purchased from Qinchengletter Ltd. Co. (Beijing, China) with purity of 99.4%.

#### 2.2.2. Animals

Prior to study initiation, the experimental protocol was reviewed and approved by the Committee on Animal Research and Ethics of Harbin Medical University (Harbin, China). Eighty-one female Sprague-Dawley (SD) rats, weighing 60–80 g, were purchased from Weitonglihua Experimental Animal Ltd. Co. (Beijing, China, Batch number SCXK (jing) 2012-0001). The animals were caged in a room maintained at 23 ± 2°C. After adaption for 1 week, rats were randomly divided into 9 groups in single cage. Each group was divided by 3*∗*3 factorial design with different dose of isoflavones and *γ*-HCH (Table 1). Isoflavones were added into diet. *γ*-HCH was dissolved with corn oil and administrated by intragastric administration.

SD rats were fed in the standard animal laboratory for 4 weeks. At the end of the experiment, rats were sacrificed after being fasted for 12 h. Body organs such as brain, liver, kidney, spleen, and the parametrial and perirenal fat pads of rats were dissected and weighed. Blood was collected from the sacrificed animals and serum was separated from the blood (3,000 rpm, 15 min) for the determination of estradiol and testosterone using radioimmunoassay and serum glucose using automatic biochemical analyzer (Hitachi 7100, Japan). The reagent kits were bought from Northern Institution of Biotechnology (Beijing, China). Histopathological examination was performed on 5 *μ*m paraffin sections with standard hematoxylin-eosin (HE) staining. The examination was performed blindly. Immunohistochemistry was performed to investigate the expression of ER*α* in uterus with ER*α* antibody from Zhongshan Technology Ltd. Co. (Beijing, China). The representative pictures were digitized by inverted microscope (Nikon Ti-S, Japan). The immunohistochemistry staining was quantified by using Image-Pro Plus software (Media Cybernetics, USA).

### 2.3. Statistics

The statistical analyses were performed with the SPSS program. Data was expressed as means ± SD. Data in this work were normally distributed determined with the Kolmogorov-Smirnov test. The data of cell culture study was analyzed with one-way ANOVA. Animal study was analyzed using a two-way ANOVA with isoflavones and OCPs treatments as factors, followed by a post hoc test with Fisher's least significant difference (LSD). *p* value < 0.05 was considered significant for all statistical analyses.

## 3. Results

### 3.1. *In Vitro*

**Figure 1:**
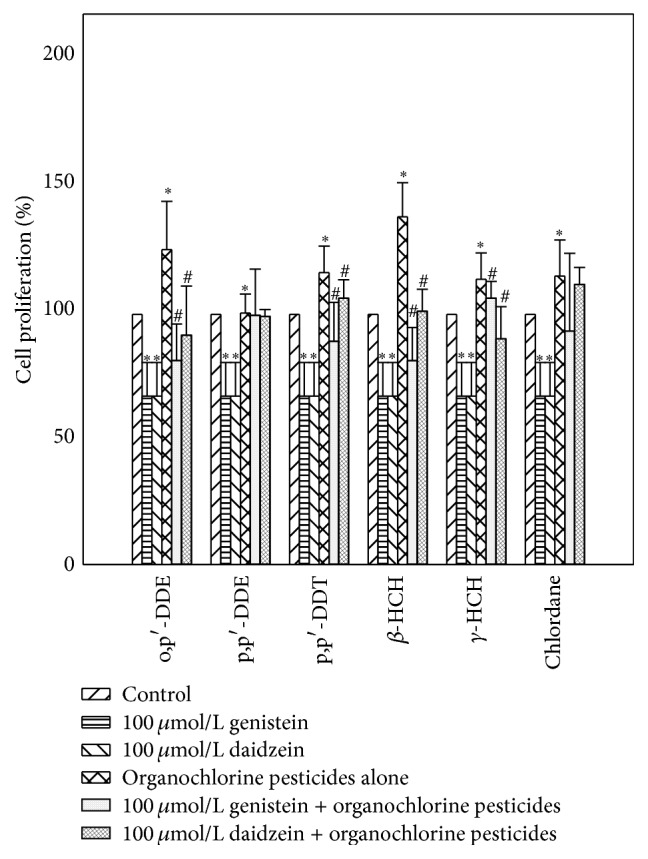
Interaction of genistein and daidzein with OCPs on proliferation rate of MCF-7 cells. ^*∗*^ *p* value < 0.05 was considered significant compared with control group; ^#^ *p* value < 0.05 was considered significant compared with organochlorine pesticides alone group.

The interaction of genistein and daidzein with OCPs in MCF-7 cells was shown in Figure 1. Compared to control group, OCPs could increase proliferation rate of MCF-7 cell line while genistein and daidzein could inhibit the growth of MCF-7 cell observably (*p* < 0.05). Compared with OCPs alone group (o,p′-DDT, p,p′-DDT, *β*-HCH, and *γ*-HCH), proliferation rate of MCF-7 cells was lower in both 100 *μ*mol/L genistein + OCPs group and daidzein + OCPs group (*p* < 0.05). Furthermore, proliferation rate of MCF-7 cells in genistein treatment group was lower than daidzein treatment group in o,p′-DDT, p,p′-DDT, and *β*-HCH group, higher in *γ*-HCH group.

### 3.2. *In Vivo*

#### 3.2.1. Effect of Isoflavones and *γ*-HCH on Body Weight

**Figure 2:**
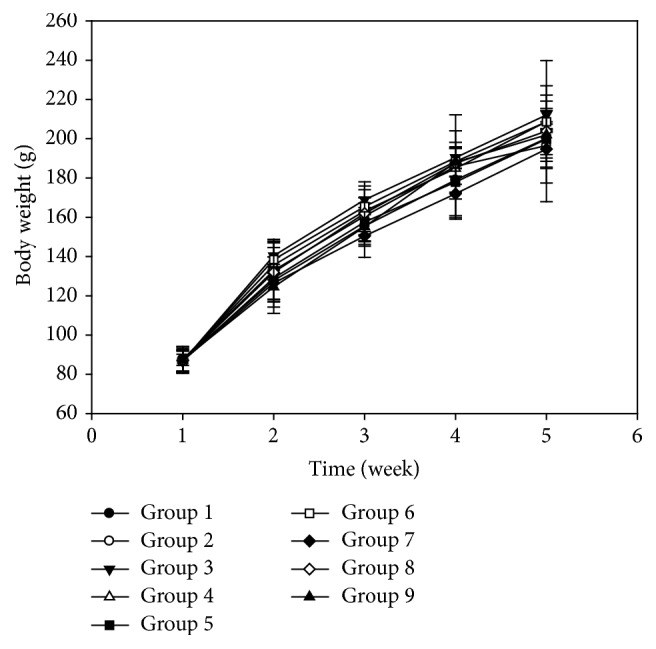
Effect of isoflavones and *γ*-HCH on body weight in female SD rats. Group 1: 900 mg/kg diet isoflavones and 8 mg/kg bw *γ*-HCH; group 2: 900 mg/kg diet isoflavones and 4 mg/kg bw *γ*-HCH; group 3: 900 mg/kg diet isoflavones and 0 mg/kg bw *γ*-HCH; group 4: 600 mg/kg diet isoflavones and 8 mg/kg bw *γ*-HCH; group 5: 600 mg/kg diet isoflavones and 4 mg/kg bw *γ*-HCH; group 6: 600 mg/kg diet isoflavones and 0 mg/kg bw *γ*-HCH; group 7: 0 mg/kg diet isoflavones and 8 mg/kg bw *γ*-HCH; group 8: 0 mg/kg diet isoflavones and 4 mg/kg bw *γ*-HCH; group 9: 0 mg/kg diet isoflavones and 0 mg/kg bw *γ*-HCH.

Effect of isoflavones and *γ*-HCH on body weight was shown in Figure 2. Rats grew normally and the body weight increased step by step from beginning of experiment to the end. Two-way ANOVA indicated that no significant differences were found in rats' body weight treated with different dosage of isoflavones and *γ*-HCH (*p* > 0.05).

#### 3.2.2. Interaction of Isoflavones and *γ*-HCH

The main effects of isoflavones and *γ*-HCH and their interaction were shown in Table 2. According to Table 2, the effect of isoflavones treatment on fasting blood glucose was significant (*p* = 0.022); that is, serum glucose levels in 600 mg/kg diet isoflavones treatment groups were increased markedly compared to 0 mg/kg diet isoflavones treatment groups (6.1 ± 0.2 mmol/L in 900 mg/kg diet treatment groups, 6.8 ± 0.2 mmol/L in 600 mg/kg diet treatment groups, and 6.2 ± 0.2 mmol/L in 0 mg/kg diet treatment groups). No main effect of *γ*-HCH and interaction effects were found in serum fasting blood glucose. In addition, the main effect of *γ*-HCH in serum estradiol and testosterone levels was prominent; that is, *γ*-HCH could reduce serum level of estradiol and testosterone notably (estradiol: 33.64 ± 2.39 pg/mL in 8 mg/kg bw, 36.45 ± 2.39 pg/mL in 4 mg/kg bw, and 45.37 ± 2.51 pg/mL in 0 mg/kg bw; testosterone: 1.45 ± 0.22 ng/mL in 8 mg/kg bw, 1.66 ± 0.22 ng/mL in 4 mg/kg bw, and 2.30 ± 0.22 ng/mL in 0 mg/kg bw). No main effect of isoflavones and interaction effects were found. Furthermore, both isoflavones and *γ*-HCH could alter spleen weight significantly. Isoflavones had a tendency of increase, while *γ*-HCH could reduce spleen weight (data not shown). Overall, there are no interactions between isoflavones and *γ*-HCH except kidney (*p* = 0.047) and liver weight (*p* = 0.033). The changes of liver weight in isoflavones 900 mg/kg diet group were different from isoflavones 600 mg/kg diet along with the increase of *γ*-HCH dose. In isoflavones 600 mg/kg diet group, liver weight was increased significantly with the increase of *γ*-HCH dose (*γ*-HCH 8 mg/kg bw group: 7.36 ± 0.69 g; *γ*-HCH 4 mg/kg bw group: 6.97 ± 1.19 g; *γ*-HCH 0 mg/kg bw group: 6.55 ± 0.84 g). However, in isoflavones 900 mg/kg diet, liver weight was highest in *γ*-HCH 8 mg/kg bw group (7.70 ± 0.52 g) and lowest in *γ*-HCH 4 mg/kg bw group (6.80 ± 0.32 g). In addition, the changes of kidney weight in *γ*-HCH 8 mg/kg bw were different from *γ*-HCH 4 mg/kg bw along with the increase of isoflavones dose. In *γ*-HCH 8 mg/kg bw group, kidney weight was highest in isoflavones 600 mg/kg diet group (900 mg/kg diet: 1.55 ± 0.14 g; 600 mg/kg diet: 1.74 ± 0.13 g; 0 mg/kg diet: 1.54 ± 0.13 g). But in *γ*-HCH 4 mg/kg bw group, kidney weight was highest in isoflavones 0 mg/kg diet group (900 mg/kg diet: 1.63 ± 0.14 g; 600 mg/kg diet: 1.63 ± 0.27 g; 0 mg/kg diet: 1.69 ± 0.18 g).

#### 3.2.3. Interaction of Isoflavones and *γ*-HCH on Uterine Morphology

**Figure 3:**
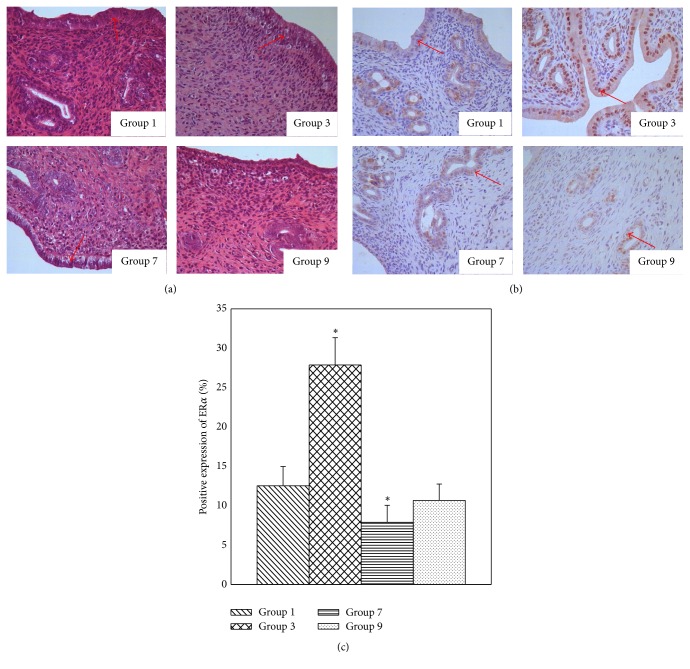
Interaction of isoflavones and *γ*-HCH on uterine morphology and expression of ER*α*. (a) showed interaction of isoflavones and *γ*-HCH on uterine morphology. Compared with control group (group 9), high columnar endometrial glandular epithelial cells were found in group 3, while low columnar endometrial glandular epithelial cells were shown in group 7. Furthermore, although high columnar endometrial glandular epithelial cells were found in group 1, the degree of hyperplasia was lower than isoflavones treatment group and higher than *γ*-HCH treatment group. (b) showed interaction of isoflavones and *γ*-HCH on expression of ER*α*. Compared to group 9, ER*α* expression was higher in group 3 (*p* ≤ 0.05), while being lower in group 7 (*p* ≤ 0.05). Moreover, ER*α* expression in group 1 was lower than group 3 and higher than group 7. (c) showed positive expression of ER*α* in immunohistochemistry. ^*∗*^ *p* value < 0.05 was considered significant compared with control group (group 9). Group 1 was treated with 900 mg/kg diet isoflavones and 8 mg/kg bw *γ*-HCH; group 3 was treated with 900 mg/kg diet isoflavones alone; group 7 was treated with 8 mg/kg bw *γ*-HCH alone; group 9 was treated with 0 mg/kg diet isoflavones and 0 mg/kg bw *γ*-HCH.

To make the comparison simpler and easier, only uterine morphology in groups 1, 3, 7, and 9 was analyzed. The interaction of isoflavones and *γ*-HCH on uterine morphology was shown in Figure 3. Compared with control group, tissue morphology was changed significantly in isoflavones treatment group and *γ*-HCH treatment group. High columnar endometrial glandular epithelial cells were found in isoflavones treatment group, while low columnar endometrial glandular epithelial cells were shown in *γ*-HCH treatment group. Furthermore, although high columnar endometrial glandular epithelial cells were found in group 1 with mixed treatment of 900 mg/kg diet isoflavones and 8 mg/kg bw *γ*-HCH, the degree of hyperplasia was lower than isoflavones treatment group and higher than *γ*-HCH treatment group (Figure 3(a)). In addition, the changes of expression of ER*α* in uterus were similar to the changes of uterine morphology (Figure 3(b)). Compared with control group, the positive expression of ER*α* in isoflavones treatment group was significantly higher (*p* < 0.001) and lower in *γ*-HCH treatment group (*p* = 0.025). The positive expression rate of ER*α* in group 1 with 900 mg/kg diet isoflavones and 8 mg/kg bw *γ*-HCH was lower than isoflavones treatment group and higher than *γ*-HCH treatment group (Figure 3(c)).

## 4. Discussion

EDCs are chemicals that may interfere with the body's endocrine system and produce adverse developmental, reproductive, neurological, and immune effects in both humans and wildlife. EDCs are derived from a wealth of sources, including OCPs, xenoestrogens, long-chain alkylphenols, and phytoestrogen. MCF-7 cells derived from human breast cancer cells, which are positive to ER and sensitive to estrogen, have been widely used to evaluate environmental estrogen and explore the development mechanism of estrogen on breast cancer occurrence [12, 13]. Genistein is a phytoestrogen with a plant-derived phenolic compound that structurally mimics the hormone 17 *β*-estradiol. Previous studies revealed that genistein could significantly inhibit the proliferation of MCF-7 cells in a dose-dependent manner. High dose of genistein could inhibit proliferation rate of MCF-7 cells [6, 12, 14]. Besides, daidzein and genistein exhibited biphasic effects (stimulatory or inhibitory) on proliferation and ER*α* expression in MCF-7 cells. That is, 1 *μ*mol/L daidzein significantly stimulates cell growth and 200 *μ*mol/L daidzein could inhibit cell proliferation [6]. In this study, we found that both 100 *μ*mol/L genistein and 100 *μ*mol/L daidzein could inhibit proliferation of MCF-7 cells. The dichlorodiphenyltrichloroethane (DDT), a known endocrine disruptor, links to animal and human disorders [15]. Payne et al. found that o,p′-DDT, p,p′-DDE, *β*-HCH, and p,p′-DDT produce proliferative effects in MCF-7 cells. Furthermore, combined effects were demonstrated by regression analyses even when each mixture component was present at levels or below its individual no-observed-effect concentration [16]. However, the research on interaction between soy isoflavones (genistein and daidzein) and OCPs was little. According to Charles et al., both* in vitro* and* in vivo*, low concentrations of the six synthetic chemicals (SCs, including o,p-DDT) failed to increase estrogenic responses which were induced by plant-derived phytoestrogens (PEs) alone.* In vitro*, interactions between high dosages of SCs and PEs were greater than mixtures of SCs in the absence of PEs [17]. In our study, soy isoflavones (genistein and daidzein) could repair the damage of OCPs; that is, isoflavones could inhibit breast cell proliferation triggered by OCPs except p,p′-DDE and chlordane.

*In vivo* test was conducted according to the results of* in vitro* experiment. We used soy isoflavones as a mixture of genistein and daidzein. Overall, isoflavones could influence blood glucose and spleen weight in female rats; *γ*-HCH could affect estradiol and testosterone levels in serum and spleen weight in high dose treatment. Furthermore, there is interaction effect on kidney weight and liver weight when treated with isoflavones and *γ*-HCH. No other effects were found in body weight, brain weight, fat weight, and so forth.

Previous study showed that soy isoflavones could significantly decrease body weight [18], while HCB, p,p′-DDE, and *β*-HCH showed quadratic associations with BMI [19]. In this study, no significant changes were found in isoflavones and OCPs treatment. Ye et al. found that both daidzein and genistein did not have a significant effect on glycemic control and insulin sensitivity over a 6-month supplementation period in Chinese women [20]. Another research showed that isoflavones mixture could lower blood glucose level in serum in high-fat diet fed C57BL/6J mice for 92 days [21]. But in this study, low dosage of isoflavones (600 mg/kg diet) could increase blood glucose level in female rats. The possible causes of differences between previous study and the current study are as follows: (1) SD rats were used in the present study instead of C57/BL6 mice, which have different absorption and ingestion for different species; (2) natural synthesis isoflavones were used as the intervention medicine, and they were different from the mixture of daidzin and glycitin used in their study. Otherwise, the interaction effect of isoflavones and *γ*-HCH on kidney weight and liver weight indicated that they could resist their side effects to produce healthy condition.

A wide number of pesticides, including OCPs, such as *γ*-HCH, may induce reproductive and developmental alterations by altering steroid hormone metabolism or binding to the estrogen/androgen receptors. Exposure to *γ*-HCH with dose of 25 mg/kg bw* in vivo* could increase absolute and relative uterus weight in F1 pups on postnatal day 22 and change vaginal patency and reduce diameters of primary oocytes at fully sexual maturity [22]. According to findings of Huang et al., both chlordane and *γ*-HCH treatment could increase estrogen, reduce testosterone, and alter morphology of the masculine appendage in shrimp [23]. In our study, estradiol and testosterone levels in serum were reduced in female rats treated with *γ*-HCH. Furthermore, the changes of uterine morphology and expression of ER*α* showed that there was interaction effect between isoflavones and *γ*-HCH.

## 5. Conclusions

In summary, high dosage of genistein and daidzein could inhibit proliferation of MCF-7 cells treated with OCPs. Besides, there are interaction effects between isoflavones and *γ*-HCH in uterine morphology and expression of ER*α*. The mechanisms of interactions between OCPs and isoflavones are not clear. Competitive inhibition effect may possibly be involved in the mechanism. Isoflavones show stronger affinity to ER, which may inhibit the combination of *γ*-HCH to ER.

## Figures and Tables

**Table 1:** Groups by 3*∗*3 factorial design with different dose of isoflavones and *γ*-HCH (*n* = 9/group).

*γ*-HCH (mg/kg bw)	Isoflavones (mg/kg diet)		
	900	600	0
8	1	4	7
4	2	5	8
0	3	6	9

Note: factorial design with 2 factors of 3 levels. Three levels of *γ*-HCH: 8 mg/kg bw, 4 mg/kg bw, and 0 mg/kg bw. Three levels of isoflavones: 900 mg/kg diet, 600 mg/kg diet, and 0 mg/kg diet.

**Table 2:** Two-way ANOVA analysis of effect of isoflavones and *γ*-HCH treatment on rats (*p* value).

	Main effect		Interaction effect
	Isoflavones	*γ*-HCH	
Blood glucose	0.022^*∗*^	0.423	0.419
Estradiol	0.159	0.003^*∗*^	0.652
Testosterone	0.749	0.020^*∗*^	0.110
Spleen weight	0.015^*∗*^	0.012^*∗*^	0.795
Kidney weight	0.529	0.566	0.047^*∗*^
Liver weight	0.283	0.050	0.033^*∗*^

^*∗*^ *p* value < 0.05 was considered significant.
